# Disability and return to work after MRI on suspicion of scaphoid fracture: Influence of MRI pathology and occupational mechanical exposures

**DOI:** 10.1371/journal.pone.0197978

**Published:** 2018-06-04

**Authors:** Lone Kirkeby, Poul Frost, Torben Bæk Hansen, Susanne Wulff Svendsen

**Affiliations:** 1 University Clinic of Hand, Hip and Knee Surgery, Regional Hospital Holstebro, Aarhus University, Holstebro, Denmark; 2 Danish Ramazzini Centre, Department of Occupational Medicine, Aarhus University Hospital, Aarhus, Denmark; 3 Danish Ramazzini Centre, Department of Occupational Medicine, Regional Hospital West Jutland–University Research Clinic, Herning, Denmark; Augusta University, UNITED STATES

## Abstract

**Objectives:**

We aimed to determine the prognosis after early MRI on clinical suspicion of scaphoid fracture, hypothesising that MRI pathology is associated with more disability and that MRI pathology and high occupational mechanical hand-arm exposures are associated with slower return to work (RTW).

**Methods:**

We conducted a follow-up study of a cohort of 469 patients, who were scanned in the period 2006 to 2010. The respondents constituted our cohort for disability analysis and the subset that was in the labour market at the time of the trauma constituted our sub-cohort for RTW analysis. Questionnaires included disability scores, job title, and lifestyle factors. Job titles were linked with a job exposure matrix to estimate occupational exposures. Register information was obtained on time until RTW. We used logistic regression analysis of disability and Cox regression analysis of time until RTW.

**Results:**

The proportion that responded was 53% (249/469) for the disability analysis and 59% (125/212) for the RTW analysis. The mean age at follow up was 43.5 years, the mean time since trauma was 4.8 years, 53% had injury of the dominant hand, and 54% had MRI pathology. Men constituted 43% of the cohort and 56% of the sub-cohort. MRI pathology was not associated with more disability [e.g., for a 'Disabilities of the Arm Shoulder and Hand'-score ≥20 the odds ratio was 0.58 (95% confidence interval 0.26–1.17)]. Patients without MRI pathology and with low occupational exposures were off work for an average of four weeks. Patients with MRI pathology or high occupational exposures were off work for twice as long time.

**Conclusion:**

MRI pathology was not associated with more disability. For patients, who were in the labour market at the time of the trauma, MRI pathology and high occupational mechanical hand-arm exposures were associated with slower RTW.

## Introduction

Early magnetic resonance imaging (MRI) is considered the golden standard in case of suspected scaphoid fracture [1–4], but the management of these patients varies [5]. Early MRI also detects radiologically occult wrist injuries other than scaphoid fractures, including bone bruising and fractures of the distal radius, other carpal bones, and metacarpal bones.

Most wrist injury patients achieve good functional outcomes, even in case of a displaced scaphoid fracture or non-union [6–9]. For wrist injury patients without MRI features of acute carpal pathology, the prognosis has not been described. Studies concerning return to work (RTW) are scarce. Higher severity of injury, pain, lower preinjury income, heavy physical job demands, and pursuit of a compensation claim have been identified as predictors of delayed RTW [10–12]. The rate of RTW has not been studied in relation to quantitative measures of occupational mechanical hand-arm exposures (forceful work, repetitive work, and work with non-neutral postures).

The aim of this study was to determine the prognosis after early MRI on suspicion of scaphoid fracture, examining the hypotheses:

1. MRI pathology is associated with more disability.
2. MRI pathology and high occupational mechanical exposures are associated with delayed RTW.

## Materials and methods

### Design and study population

We conducted a follow up study within a cohort of 597 consecutive patients, who underwent early MRI on suspicion of scaphoid fracture as part of the standard examination and treatment algorithm of the emergency department, 2006–2010 [13]. Patients with MRI-verified scaphoid fracture or bone bruise were treated with a scaphoid cast (plaster cast with thumb extension), whereas patients with MRI-verified fracture of the distal radius were treated with a dorsal wrist splint. Other bone bruises (in the distal radius or carpal bones other than the scaphoid) were treated with a dorsal wrist splint without thumb extension. One patient with an acute ligament injury had surgery. Other patients with MRI-verified soft tissue injuries and patients whose MRI showed no acute pathology were treated with a soft elastic bandage, which they were encouraged to discontinue to use within a couple of weeks.

Patients were included in the present study if they were ≥18 years old at the time of follow up (we included patients who were <18 years at the time of their wrist trauma because they would be informative for the disability analysis. Regarding age restriction for the RTW analysis, please see below). In case of more than one wrist trauma with MRI (n = 4), only the first was included in the present study. Information on vital status, emigration, and current addresses was obtained from the Danish Civil Registration System [14]. Patients who had died or emigrated or had unknown address were excluded. In 2013 we sent a follow up questionnaire by mail with a maximum of two reminders after 3 and 6 weeks. For both respondents and non-respondents, we obtained information on labour market attachment from the Danish National Register on Public Transfer Payments [15, 16]. The respondents constituted our cohort for disability analysis (the *DASH/PRWE/WAS cohort*–the abbreviations are explained below in the section on outcome measures). The subset that was ≥18 and ≤63 years old and in the labour market (employed/unemployed) at the time of the trauma constituted our sub-cohort for RTW analysis (the *RTW sub-cohort*).

### Potential prognostic factors

For research purposes, findings indicating acute carpal pathology were categorised as fractures, bone bruise, and soft tissue injury.

Questionnaire information was collected on job title at the time of injury. The job title was linked with a job exposure matrix (JEM) to obtain estimates of occupational mechanical hand-arm exposures for each patient. The JEM was originally constructed for a study of ulnar neuropathy; details have been provided elsewhere [17]. In brief, the hand-arm JEM is based on the mean of five experts’ ratings of occupational mechanical exposures for 806 occupational titles, which were divided into 169 groups of jobs with expected homogeneous exposure profiles. The experts rated the job groups with respect to forceful work using a five point scale (force-score: 0 = light, 1 = somewhat hard, 2 = hard, 3 = very hard, and 4 = near maximal) [18]. They also rated the mean number of hours per day with repetitive movements of the elbow or wrist (≥4 movements per minute, excluding computer use) and the mean number of hours per day with non-neutral postures of the elbow (flexion >100^o^, or ≥near maximal pronation or supination) and/or wrist (>5^o^ radial deviation, >10^o^ ulnar deviation or >15^o^ palmar/dorsal flexion). The questionnaire responses included eight job titles, which could not be converted to occupational titles in the original JEM; instead, we used JEM estimates for occupational titles with similar exposure profiles.

Age and sex were registered, and we collected questionnaire information on handedness, height, weight, and smoking status. Body mass index (BMI) was calculated as weight divided by height squared (kg/m^2^). We used BMI and smoking status at follow up as proxies for the corresponding baseline information. For the RTW sub-cohort, we obtained information on sick leave >2 weeks during the year before the trauma from the Danish National Register on Public Transfer Payments [15] so that we could control for the patients’ habitual sickness absence level. Using the same register, we estimated the patients’ education level based on unemployment insurance fund membership in the year before the trauma [19, 20]. Education level was categorised into higher or medium-level education, vocational education and training, and low education level including cash benefit, which is part of the lowest level of the social safety net in Denmark [19, 20]. We used education level ad an indicator for socioeconomic status [20].

### Outcome measures

The questionnaire included the Disabilities of the Arm, Shoulder and Hand (DASH) and the Patient-Rated Wrist Evaluation (PRWE), which have been validated and translated into Danish [21, 22]. The DASH is a 30-item questionnaire designed to measure disability and symptoms of both upper limbs together [23, 24]. The PRWE is a 15-item questionnaire which measures side-specific pain and function in wrist disorders [25, 26]. Both scores range from 0 (best) to 100 (worst). When calculating the DASH score, up to three missing values were replaced by the mean value of the other items [23]. When calculating the PRWE score, up to one item from each of the two subscores (pain and function) was replaced by the mean value of the other items of the subscore (http://srs-mcmaster.ca/wp-content/uploads/2015/05/English-PRWE-User-Manual.pdf). We dichotomised both scores at ≥20 to define a high DASH and a high PRWE as our disability outcome measures. The patients were also asked to estimate their current work ability compared with their lifetime best using the Work Ability Score (WAS), which ranges from 0 (completely unable to work) to 10 (work ability at its best) [27, 28]. We used a WAS <8 as a measure of work disability. The three cut off values were chosen so that around one third of the patients were in the high categories. For DASH and PRWE, the cut off values for disability further reflected general population mean scores (around 9 for DASH [29] and around 7 for PRWE [30]) with addition of estimated minimal clinically relevant differences (around 12 for DASH [31] and around 14 for PRWE [32]). For WAS, the cut off was set between excellent (10 points) and good (8–9 points) work ability on the one hand and moderate (6–7 points) and poor (0–5 points) work ability on the other [33]. Poor and moderate WAS values have been reported to predict disability pension and long-term sickness absence among Finnish employees [34].

For the RTW sub-cohort, we further obtained data on sickness absence from the Danish National Register on Public Transfer Payments. RTW was defined as the first completion of a period of four consecutive weeks on the labour market within 104 weeks after the wrist injury [35]. We asked patients, who had a job at baseline, if they had resumed the same job after the trauma.

### Statistical methods

For the DASH/PRWE/WAS cohort, we compared questionnaire respondents and non-respondents with respect to age, sex, MRI pathology (no/yes), and labour market attachment. We used uni- and multivariable logistic regression to analyse personal characteristics as predictors of disability (DASH ≥20, PRWE ≥20, and WAS <8, respectively). The multivariable analyses were performed with mutual adjustment for all variables in the model, i.e. continuous age at trauma, sex, BMI (<25/25-<30/≥30 kg/m^2^), smoking (never/ex-/current), injury of dominant hand (no/yes), time since trauma (≤4 years/>4 years), and MRI pathology (no/yes). A test for trend was based on continuous BMI. In a supplementary analysis, we included age as a dichotomous variable (<18/≥18) to account for a potential negative prognostic influence of being skeletally immature at the time of the trauma.

For the RTW sub-cohort, predictors of time until RTW were analysed using uni- and multivariable Cox regression. Participants were followed until RTW, death, emigration, retirement, becoming 65 years old, or end of follow up after 104 weeks, whichever came first. The full model comprised the personal characteristics mentioned above, forceful work (force-score: 0/>0-<1/≥1), repetitive work (0/>0-<2.5/≥2.5 hours/day), non-neutral postures (<1/≥1-<2/≥2 hours/day), education level (higher or medium-level/vocational education and training/low level), and sick leave >2 weeks during the year before the trauma (no/yes). Only forceful work was included when adjusting MRI findings and personal characteristics for occupational mechanical exposures, for one thing because of collinearity between the exposures, for another because forceful work was the only exposure with statistically significant results. We used multivariable Cox regression to compare RTW for respondents and non-respondents adjusting for variables which did not rely on self-report (age, sex, education level, sick-leave, and MRI pathology). Cox regression analyses yielded hazard ratios (HR) with 95% confidence intervals (CIs) for RTW. Please, note that a HR below one indicates slower RTW. The proportional-hazards assumption was tested using Schoenfeld residuals. For forceful work, these analyses were supplemented by test for trend using the force-score categories as a continuous variable. Based on the multivariable Cox regression models, we plotted the survivor function in relation to MRI pathology and force-score categories, respectively.

### Ethics

The Danish Data Protection Agency approved the study (j.nos. 1-16-02-258-12 and 1-16-02-74-17). In Denmark, questionnaire- and register-based studies do not require approval by committees on biomedical research or patient consent. Two of the authors were members of the physician staff at the department at the time of the examinations. We removed Civil Personal Register numbers from the analysis data set after linkage of data.

## Results

**Fig 1:**
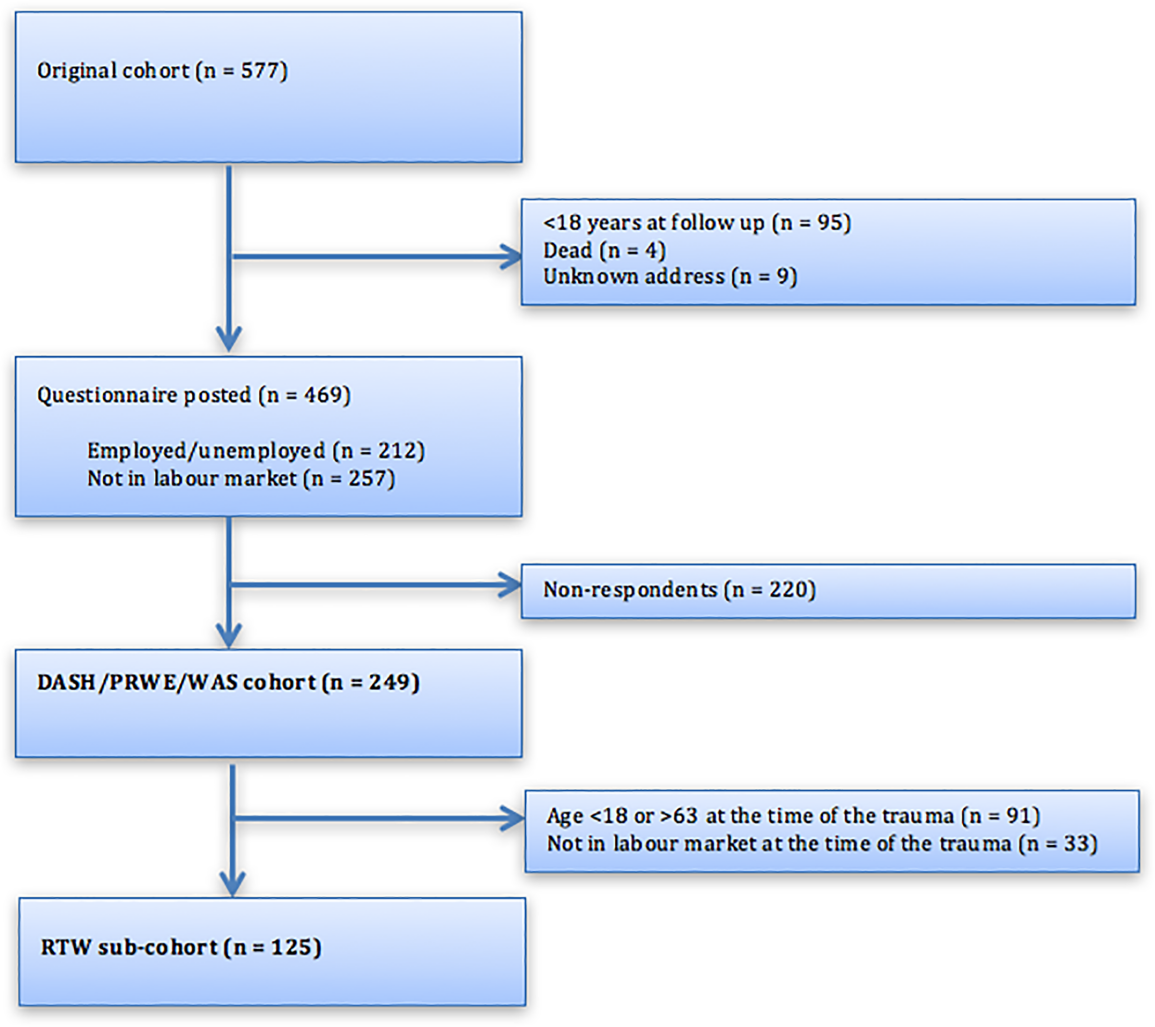
Flow chart of the DASH/PRWE/WAS cohort and the RTW sub-cohort. Abbreviations: DASH = Disabilities of the Arm, Shoulder and Hand; PRWE = Patient-Rated Wrist Evaluation; RTW = return to work; WAS = Work Ability Score.

Fig 1 presents the flow chart. For the DASH/PRWE/WAS cohort, the mean time since trauma was 4.8 years. The proportion that responded was 53% (249/469). Respondents were on average 11.8 years older than non-respondents at the time of the trauma (mean age 38.7 years (SD 20.0) and 26.9 years (SD 15.4), respectively), they were more often female (56.6% of respondents, 43.4% of non-respondents), and the percentage with MRI pathology was higher (57.7% of respondents, 42.3% of non-respondents). Table 1 shows personal characteristics of the questionnaire respondents. The age range at the time of the trauma was 12 to 85 years, with 56 respondents (22%) under the age of 18.

**Table 1:** Personal characteristics of the DASH/PRWE/WAS cohort and the RTW sub-cohort.

Personal characteristics	DASH/PRWE/WAS cohort (n = 249)		RTW sub-cohort (n = 125)	
Age at trauma (years), mean (SD)	38.7	(20.0)	40.2	(13.8)
Age at follow up (years), mean (SD)	43.5	(19.7)	45.0	(13.8)
Sex, n (%)				
Male	108	(43.4)	70	(56.0)
Female	141	(56.6)	55	(44.0)
Body mass index (kg/m^2^), n (%)				
<25	111	(44.6)	52	(41.6)
25-<30	91	(36.6)	49	(39.2)
≥30	35	(14.1)	20	(16.0)
Missing	12	(4.8)	4	(3.2)
Smoking, n (%)				
Never smoker	105	(42.2)	52	(41.6)
Ex-smoker	71	(28.5)	40	(32.0)
Current smoker	61	(24.5)	29	(23.2)
Missing	12	(4.8)	4	(3.2)
Injury of dominant hand, n (%)				
No	116	(46.6)	57	(45.6)
Yes	133	(53.4)	68	(54.4)
Time since trauma, n (%)				
≤4 years	105	(42.2)	49	(39.2)
>4 years	144	(57.8)	76	(60.8)
MRI pathology, n (%)				
No	114	(45.8)	65	(52.0)
Bone bruise	61	(24.5)	27	(21.6)
Fracture	62	(24.9)	25	(20.0)
Other acute pathology	12	(4.8)	8	(6.4)

Percentages do not always add up to 100 due to rounding.

Abbreviations: DASH = Disabilities of the Arm, Shoulder and Hand; MRI = magnetic resonance imaging; PRWE = Patient-Rated Wrist Evaluation; RTW = return to work; WAS = Work Ability Score.

The proportion who responded in the RTW sub-cohort was 59% (125/212), and respondents and non-respondents did not differ with respect to time until RTW; when we compared non-respondents to respondents, the adjusted HR was 1.01 (95% CI 0.74–1.36). For the RTW sub-cohort, occupational exposures and education level are cross-tabulated in Table 2. The most frequent occupational titles among male respondents were meat and fish processing machine operators, heavy truck and lorry drivers, and armed forces, which together covered 21% of this group; the same occupational titles prevailed in the group with a force-score ≥1. The most frequent occupational titles among female respondents were shop sales persons and demonstrators, pre-primary education teaching associate professionals, and office clerks, which together covered 24% of this group; the most frequent occupational titles with a force-score ≥1 were shop sales persons and demonstrators, pre-primary education teaching associate professionals, and cleaners.

**Table 2:** Occupational mechanical exposures and education level of the RTW sub-cohort.

Occupational mechanical exposures	Education level		
	Higher or medium-level n = 42	Vocational education and training n = 45	Low level n = 38
Forceful work (force-score)			
0	33.3	15.6	5.3
>0-<1	33.3	15.6	26.3
≥1	28.6	62.2	52.6
Missing	4.8	6.7	15.8
Repetitive work (hours/day)			
0	57.1	26.7	47.4
>0-<2.5	28.6	15.6	18.4
≥2.5	9.5	51.1	18.4
Missing	4.8	6.7	15.8
Work with non-neutral postures (hours/day)			
<1	64.3	26.7	39.5
≥1-<2	19.1	15.6	29.0
≥2	11.9	51.1	15.8
Missing	4.8	6.7	15.8

Values in cells are percentages of patients in the RTW sub-cohort. Percentages do not always add up to 100 due to rounding.

Abbreviation: RTW = return to work.

In total, 235 participants provided sufficient information to calculate DASH and PRWE scores. Table 3 shows disability in relation to MRI findings and personal characteristics. Contrary to what we expected, MRI pathology tended to predict lower odds of disability, but without reaching statistical significance. This tendency was found for both bone bruise and fracture (results not shown). Higher age and female sex were predictors of disability, and current smokers had higher odds of disability than ex- and never smokers. There was a tendency towards higher odds of disability with increasing BMI (test for trend, DASH: P = 0.046, PRWE: P = 0.062). DASH and PRWE scores ≥20 were not related to trauma to the dominant hand and time since trauma.

**Table 3:** Disability in terms of DASH and PRWE scores ≥20 in relation to MRI findings and personal characteristics (n = 249).

	DASH ≥20					PRWE ≥20				
	Prevalence (%)	OR _crude_	95% CI	OR _adjusted_ ^a^	95% CI	Prevalence (%)	OR _crude_	95% CI	OR _adjusted_ ^a^	95% CI
MRI pathology										
No	33.3	1.00		1.00		40.4	1.00		1.00	
Yes	24.2	0.64	0.36–1.13	0.58	0.29–1.17	31.8	0.69	0.40–1.17	0.73	0.38–1.38
Age (for an increment of 1 year)		1.01	1.00–1.03	1.02	1.00–1.04		1.01	0.99–1.02	1.02	1.00–1.03
Sex										
Male	14.6	1.00		1.00		28.9	1.00		1.00	
Female	39.4	3.81	1.99–7.41	5.01	2.41–10.71	41.2	1.73	1.00–2.99	2.17	1.17–4.04
Body mass index (kg/m^2^)										
<25	25.9	1.00		1.00		34.0	1.00		1.00	
25-<30	29.4	1.19	0.63–2.25	1.43	0.70–2.92	32.6	0.94	0.51–1.72	1.09	0.57–2.09
≥30	35.3	1.56	0.68–3.55	2.07	0.80–5.36	48.6	1.84	0.85–3.99	2.23	0.94–5.30
Smoking										
Never smoker	25.7	1.00		1.00		33.0	1.00		1.00	
Ex-smoker	22.5	0.84	0.41–1.71	0.79	0.36–1.73	28.6	0.81	0.42–1.57	0.82	0.40–1.66
Current smoker	41.8	2.07	1.03–4.16	3.57	1.56–8.15	51.9	2.19	1.11–4.29	3.31	1.56–7.01
Injury of dominant hand										
No	29.1	1.00		1.00		35.4	1.00		1.00	
Yes	28.0	0.95	0.54–1.67	1.36	0.70–2.64	36.1	0.97	0.57–1.66	1.21	0.66–2.22
Time since trauma										
≤4 years	27.4	1.00		1.00		36.3	1.00		1.00	
>4 years	30.0	1.14	0.64–2.01	0.58	0.29–1.17	35.0	0.95	0.55–1.62	0.98	0.53–1.83

Abbreviations: CI = confidence interval; DASH = Disabilities of the Arm, Shoulder and Hand; MRI = magnetic resonance imaging; OR = odds ratio; PRWE = Patient-Rated Wrist Evaluation.

^a^ Mutually adjusted for all variables in the table.

WAS was available for 240 participants. As seen in Table 4, current smoking and a high BMI predicted work disability. Again, MRI pathology tended to predict lower odds, but without reaching statistical significance. When we included age as a dichotomous variable, the adjusted OR for DASH was 0.77 (95% CI 0.33–1.58) for age <18 years old at the time of the trauma. The corresponding ORs for PRWE and WAS were 0.89 (95% CI 0.40–1.65) and 0.78 (95% CI 0.37–1.64), respectively. Thus, skeletal immaturity did not seem to be a negative prognostic factor.

**Table 4:** Work disability in terms of WAS <8 in relation to MRI findings and personal characteristics (n = 249).

	Prevalence (%)	OR _crude_	95% CI	OR _adjusted_ ^a^	95% CI
MRI pathology					
No	36.0	1.00		1.00	
Yes	26.4	0.64	0.37–1.10	0.62	0.32–1.21
Age (for an increment of 1 year)		1.01	0.99–1.03	1.02	1.01–1.04
Sex					
Male	25.5	1.00		1.00	
Female	35.1	1.58	0.90–2.78	1.77	0.94–3.32
Body mass index (kg/m^2^)					
<25	28.4	1.00		1.00	
25-<30	28.4	1.00	0.54–1.86	1.06	0.54–2.07
≥30	45.7	2.12	0.97–4.64	2.44	1.02–5.84
Smoking					
Never smoker	28.9	1.00		1.00	
Ex-smoker	22.1	0.70	0.34–1.42	0.63	0.29–1.36
Current smoker	46.7	2.16	1.11–4.18	3.22	1.54–6.75
Injury of dominant hand					
No	31.6	1.00		1.00	
Yes	30.2	0.94	0.54–1.62	1.18	0.64–2.20
Time since trauma					
≤4 years	31.7	1.00		1.00	
>4 years	29.7	0.91	0.52–1.59	1.00	0.52–1.90

Abbreviations: CI = confidence interval; MRI = magnetic resonance imaging; OR = odds ratio; WAS = Work Ability Score.

^a^ Mutually adjusted for all variables in the table.

Table 5 presents the results regarding time until RTW for the RTW sub-cohort; 112 participants could be included in the fully adjusted model. MRI pathology and forceful work predicted slower RTW (test for trend for forceful work, P = 0.048) with around twice as long time off work for patients with MRI pathology or a high force-score, and almost four times as long time off work for those with a combination of these two factors (the reciprocal of the product of the adjusted HR for MRI pathology and a high force-score, 1/(0.55x0.48) = 3.8). To evaluate if this model-based estimate was in accordance with the actual data, we calculated the adjusted HR for MRI pathology combined with a high force-score, which yielded a result of 0.29 (95% CI 0.12–0.68). Based on the reciprocal value, patients with this combination were off work 3.44 (95% CI 1.47–8.88) times longer than patients without MRI pathology and without a high force-score. Patients without MRI pathology and without a high force-score were off work for an average of 4 weeks. Repetitive work and work with non-neutral postures were negative prognostic factors as well, but these tendencies did not reach statistical significance. Sick leave >2 weeks during the year before the trauma predicted slower RTW. In total, 93% (116/125) of the patients returned to work during follow up, i.e. within 104 weeks, and 91% (114/125) resumed the same job.

**Table 5:** Observation time, numbers returned to work, incidence rate of return to work, and crude and adjusted hazard ratios for return to work with 95% confidence intervals in relation to MRI findings, personal characteristics, and occupational mechanical exposures (n = 125). A hazard ratio below one indicates *slower* return to work.

	Person-weeks of observation	Numbers returned to work	Incidence rate per 100 person-weeks	HR _crude_	95% CI	HR _adjusted_ ^a^	95% CI
**MRI findings**							
MRI pathology							
No	422	61	14.5	1.00		1.00	
Yes	691	55	8.0	0.58	0.39–0.86	0.48	0.29–0.80
**Personal characteristics**							
Age at trauma	-	-	-	0.99	0.98–1.01	1.00	0.98–1.02
Sex							
Male	545	51	9.4	1.00		1.00	
Female	568	65	11.4	1.06	0.73–1.53	0.78	0.47–2.33
Smoking							
Never smoker	415	48	11.6	1.00		1.00	
Ex-smoker	402	37	9.2	0.84	0.55–1.30	0.79	0.47–1.34
Current smoker	280	27	9.6	0.74	0.46–1.20	0.80	0.46–1.39
Body mass index (kg/m^2^)							
<25	362	47	13.0	1.00		1.00	
25-<30	511	46	9.0	0.86	0.57–1.29	0.97	0.60–1.56
≥30	224	19	8.5	0.95	0.55–1.61	0.79	0.43–1.45
Injury of dominant hand							
No	467	54	11.6	1.00		1.00	
Yes	646	62	9.6	1.02	0.71–1.47	0.98	0.64–1.51
Education level							
Higher or medium-level	391	40	10.2	1.00		1.00	
Vocational education and training	444	41	9.2	0.93	0.60–1.44	1.22	0.73–2.02
Low level	278	35	12.6	1.04	0.66–1.64	1.22	0.70–2.13
Sick-leave >2 weeks during the year before the trauma							
No	606	93	15.3	1.00		1.00	
Yes	507	23	4.5	0.46	0.28–0.75	0.47	0.26–0.87
**Occupational mechanical exposures**							
Forceful work (force-score)							
0	101	23	22.8	1.00		1.00	
>0-<1	224	28	12.5	0.65	0.37–1.14	0.67	0.36–1.25
≥1	673	55	8.2	0.56	0.33–0.93	0.55	0.30–0.99
Repetitive work (hours/day)							
0	316	51	16.1	1.00		1.00	
>0-<2.5	265	22	8.3	0.81	0.48–1.34	0.68	0.39–1.17
≥2.5	417	33	7.9	0.68	0.43–1.07	0.75	0.42–1.33
Work with non-neutral postures (hours/day)							
<1	336	51	15.2	1.00		1.00	
≥1-<2	374	21	5.6	0.66	0.39–1.10	0.55	0.29–1.02
≥2	288	34	11.8	0.84	0.54–1.30	0.86	0.48–1.53

Abbreviations: CI = confidence interval; HR = hazard ratio; MRI = magnetic resonance imaging.

^a^ MRI findings and personal characteristics were mutually adjusted for each other and for forceful work. Occupational mechanical exposures were analysed one at a time and adjusted for MRI findings and personal characteristics.

**Fig 2:**
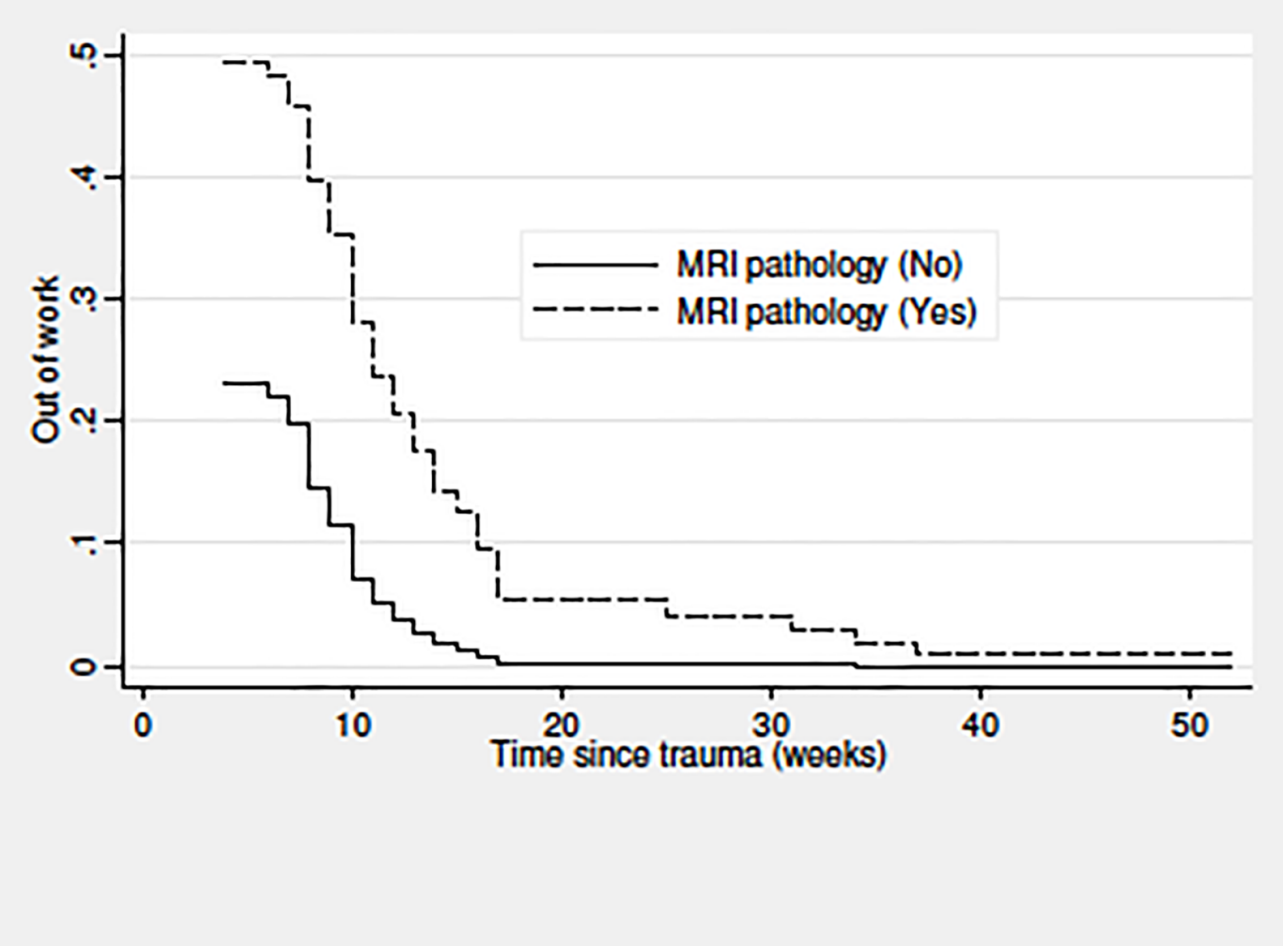
Time (weeks) until return to work for at least four weeks in relation to MRI findings among patients examined by early MRI on suspicion of scaphoid fracture (n = 125). Plot from multivariable Cox proportional hazard analyses; the Y-axis shows the probability (decimal numbers) of being out of work. Abbreviation: MRI = magnetic resonance imaging.

**Fig 3:**
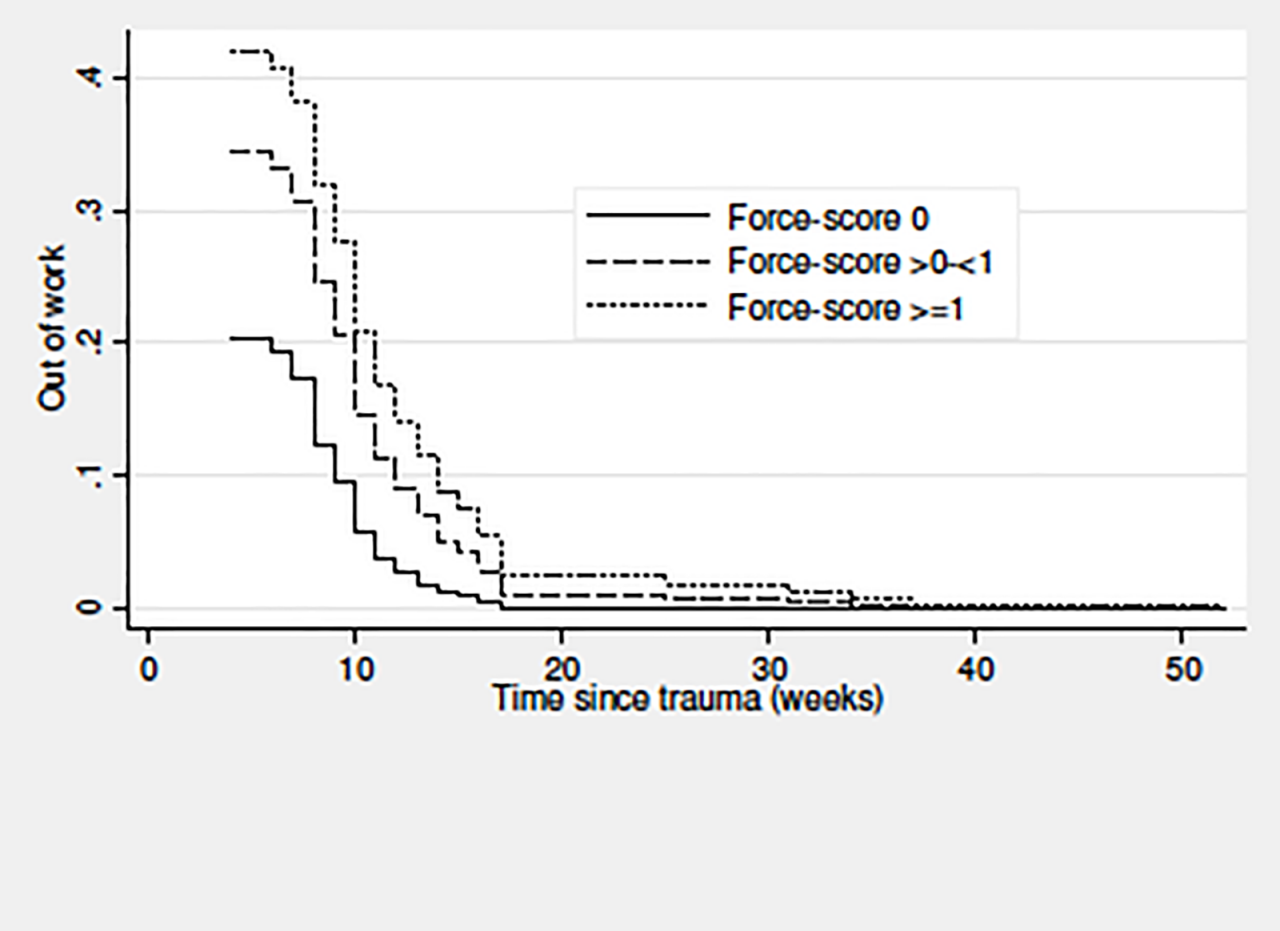
Time (weeks) until return to work for at least four weeks in relation to forceful work (force-score) among patients examined by early MRI on suspicion of scaphoid fracture (n = 125). Plot from multivariable Cox proportional hazard analyses; the Y-axis shows the probability (decimal numbers) of being out of work. Abbreviation: MRI = magnetic resonance imaging.

Figs 2 and 3 illustrate time until RTW in relation to MRI pathology (Fig 2) and forceful work (Fig 3). The differences between the compared groups were evident within one year after the trauma. Patients with MRI pathology returned to work more slowly than patients without MRI pathology (Fig 2). A higher force-score also predicted slower RTW (Fig 3).

## Discussion

This follow up study of patients, who had early MRI on clinical suspicion of scaphoid fracture, showed that MRI pathology was not associated with more disability, whereas current smoking and a high BMI were. For patients, who were employed at the time of the trauma, MRI pathology and high occupational mechanical exposures (in particular a high force-score) were associated with slower RTW.

A limitation of the study was the relatively low proportion, who responded. In the DASH/PRWE/WAS cohort, non-respondents were younger, more often male, and more often had normal MRI findings. This may have led to a negatively biased picture of the overall prognosis with respect to disability. Particularly, we may have overestimated the odds of disability for patients with normal MRI findings to the degree that non-participation reflected less disability and thereby less incentive to respond. This source of bias may be part of the explanation why MRI pathology was not associated with more disability. Lack of immobilisation in case of contusion or sprain in the absence of MRI pathology might be another part of the explanation. In the RTW sub-cohort, we had access to several potential prognostic factors, which did not rely on self-report. Thorough analysis did not reveal signs of non-response bias. In particular, respondents and non-respondents did not differ with respect to time until RTW.

DASH and PRWE are validated measures of disability related to the upper extremities. We included a low WAS as a measure of work disability with a supposedly closer link to the RTW-process. This single-item measure of work ability has been advocated as a valid way of assessing the status and progress of work ability [27, 28] and has been found to predict future sick leave [27] and RTW [36]. The present study also benefited from register information on sick leave [15, 16] and from independent information on occupational mechanical exposures from a JEM. The use of JEM-based exposure estimates precluded inflation of our results by recall bias, which could have resulted from overestimation of occupational mechanical exposures among patients with symptoms/disability if we had used self-reported exposure measures. The JEM has shown good predictive validity in a study of ulnar neuropathy [37].

Relevant cut off values to define disability depend on the purpose. We substantiated the clinical relevance of the cut offs that we chose based on reported general population means and minimal clinically important differences for DASH and PRWE, which suggested that patients in the high categories had clinically importantly elevated DASH and PRWE scores compared to background values. On the other hand, the cut offs may be considered relatively low when compared to disability levels among patients with more severe conditions. E.g., we have previously conducted a follow-up study among patients, who were examined by nerve conduction studies on suspicion of ulnar neuropathy (median follow-up 4 years) [37]. In that study, we found mean DASH scores of around 23 among the patients as compared to mean scores of around 10 among their matched population referents, whereas preoperative scores of around 30 have been reported for patients with ulnar neuropathy and scores of 55 to 60 have been reported for brachial plexus injury [37]. Likewise, a study, which provided normative data for PRWE, found background values of 6.6 for participants with no history of wrist/hand fracture or surgery, 13.6 for patients with wrist/hand fracture or surgery more than 1 year ago, and 26.7 for participants who reported that they were unfit for work [30], while a follow-up study among patients with distal radius fractures showed that the mean PRWE scores declined from 74 at baseline, through 42 at two months and 26 at three months to 19 at six months [26]. Regarding WAS, mean values of around 8 have been reported among non-sicklisted workers [38], against mean values of around 6 at baseline among workers who experienced long-term sickness absence during 4 years of follow up [39] and around 4 in long-term sicklisted female workers [40], of whom only 4% had a WAS >8 [27]. Furthermore, WAS values <8 have been reported to predict disability pension and long-term sickness absence (cf. the methods section) [34]. Since most wrist injury patients have a good prognosis (cf. the introduction), we chose relatively low but still clinically relevant cut offs to define disability.

In agreement with our hypothesis, the presence of MRI pathology predicted slower RTW. We expected this due to the pathological condition per se and the resulting immobilisation. Also in agreement with our hypothesis, high occupational exposures predicted slower RTW. The tendency reached statistical significance for forceful work only, but the same tendency was seen for repetitive work and work with non-neutral postures. Forceful work predicted slower RTW, even if there was no MRI pathology. This could be expected because patients might well experience symptoms (e.g. due to MRI-negative contusion or sprain), which would necessitate relatively long periods of sick leave in case of a job with a high force-score.

Body weight and tobacco smoking were maybe underestimated by the respondents. However, overestimation of body weight and smoking was probably rare so that participants with a high BMI or a status as current smokers were most likely correctly classified, and any reporting bias was probably non-differential, i.e. independent of the outcomes under study. Accordingly, the risk estimates for a high BMI and current smoking can be expected to be close to those that would have been found using measured information [41]. We found that smoking and a high BMI were related to disability. Smoking has been identified as a significant risk factor for non-union of the scaphoid bone [42, 43] and for non-union of fractures overall [44], but it is unclear whether smoking influences healing after minor wrist trauma. Other studies have not identified a high BMI as a risk factor for fracture-healing complications [45, 46], but this does not preclude an association with disability. In a study of ulnar neuropathy and ulnar neuropathy-like symptoms, we found that both smoking and a high BMI were related to disability as measured by DASH [37].

The age and sex distribution of the patients in the original cohort was similar to what has been reported in other studies [3, 4]. Our findings should be generalisable to other countries that apply a treatment algorithm like ours, although our findings concerning RTW may best be generalised to countries that also have similar working conditions and social welfare systems to those in Denmark.

Our results suggest that orthopaedic surgeons should ask their patients about their occupational exposures as a basis for conveying as realistic expectations as possible regarding the time required off work post-injury; this seems relevant even for wrist injury patients without MRI pathology. Patients with high occupational exposures can expect prolonged sick-leave after wrist trauma, particularly when MRI pathology is present, but also when MRI pathology is absent.

In conclusion, MRI pathology was not associated with more disability. For patients, who were in the labour market at the time of the trauma, MRI pathology and high occupational mechanical hand-arm exposures were associated with slower RTW.

## References

[1] Breitenseher MJ, Metz VM, Gilula LA, Gaebler C, Kukla C, Fleischmann D, et al Radiographically occult scaphoid fractures: value of MR imaging in detection. Radiology. 1997;203(1):245–50. doi: 10.1148/radiology.203.1.9122402 . 9122402 10.1148/radiology.203.1.9122402

[2] Hansen TB, Petersen RB, Barckman J, Uhre P, Larsen K. Cost-effectiveness of MRI in managing suspected scaphoid fractures. J Hand Surg Eur Vol. 2009;34(5):627–30. doi: 10.1177/1753193409105322 . 19687072 10.1177/1753193409105322

[3] Khalid M, Jummani ZR, Kanagaraj K, Hussain A, Robinson D, Walker R. Role of MRI in the diagnosis of clinically suspected scaphoid fracture: analysis of 611 consecutive cases and literature review. Emerg Med J. 2010;27(4):266–9. doi: 10.1136/emj.2008.058750 . 20385675 10.1136/emj.2008.058750

[4] McCullough NP, Smith FW, Cooper JG. Early MRI in the management of the clinical scaphoid fracture. Eur J Emerg Med. 2011;18(3):133–6. doi: 10.1097/MEJ.0b013e32833edb59 . 20838220 10.1097/MEJ.0b013e32833edb59

[5] Smith JE, House RH, Gallagher J, Phillips A. The management of suspected scaphoid fractures in English hospitals: a national survey. Eur J Emerg Med. 2016;23(3):190–3. doi: 10.1097/MEJ.0000000000000228 . 25460813 10.1097/MEJ.0000000000000228

[6] Euler S, Erhart S, Deml C, Kastenberger T, Gabl M, Arora R. The effect of delayed treatment on clinical and radiological effects of anterior wedge grafting for non-union of scaphoid fractures. Arch Orthop Trauma Surg. 2014;134(7):1023–30. doi: 10.1007/s00402-014-2007-7 . 24823907 10.1007/s00402-014-2007-7

[7] Megerle K, Keutgen X, Muller M, Germann G, Sauerbier M. Treatment of scaphoid non-unions of the proximal third with conventional bone grafting and mini-Herbert screws: an analysis of clinical and radiological results. J Hand Surg Eur Vol. 2008;33(2):179–85. doi: 10.1177/1753193408087030 . 18443060 10.1177/1753193408087030

[8] Bedi A, Jebson PJ, Hayden RJ, Jacobson JA, Martus JE. Internal fixation of acute, nondisplaced scaphoid waist fractures via a limited dorsal approach: an assessment of radiographic and functional outcomes. J Hand Surg Am. 2007;32(3):326–33. doi: 10.1016/j.jhsa.2007.01.002 . 17336838 10.1016/j.jhsa.2007.01.002

[9] Bae DS, Gholson JJ, Zurakowski D, Waters PM. Functional outcomes after treatment of scaphoid fractures in children and adolescents. J Pediatr Orthop. 2016;36(1):13–8. doi: 10.1097/BPO.0000000000000406 . 25658179 10.1097/BPO.0000000000000406

[10] Johns AM. Time off work after hand injury. Injury. 1981;12(5):417–24. . 7263053 10.1016/0020-1383(81)90015-2

[11] Wong JY. Time off work in hand injury patients. J Hand Surg Am. 2008;33(5):718–25. doi: 10.1016/j.jhsa.2008.01.015 . 18590855 10.1016/j.jhsa.2008.01.015

[12] Shi Q, Sinden K, MacDermid JC, Walton D, Grewal R. A systematic review of prognostic factors for return to work following work-related traumatic hand injury. J Hand Ther. 2014;27(1):55–62; quiz doi: 10.1016/j.jht.2013.10.001 . 24268193 10.1016/j.jht.2013.10.001

[13] Kirkeby L, Kairelyte V, Hansen TB. Early magnetic resonance imaging in patients with a clinically suspected scaphoid fracture may identify occult wrist injuries. J Hand Surg Eur Vol. 2013;38(5):571–2. doi: 10.1177/1753193412471008 . 23266950 10.1177/1753193412471008

[14] Schmidt M, Pedersen L, Sørensen HT. The Danish Civil Registration System as a tool in epidemiology. Eur J Epidemiol. 2014;29(8):541–9. doi: 10.1007/s10654-014-9930-3 . 24965263 10.1007/s10654-014-9930-3

[15] Hjøllund NH, Larsen FB, Andersen JH. Register-based follow-up of social benefits and other transfer payments: accuracy and degree of completeness in a Danish interdepartmental administrative database compared with a population-based survey. Scand J Public Health. 2007;35(5):497–502. doi: 10.1080/14034940701271882 . 17852980 10.1080/14034940701271882

[16] Stapelfeldt CM, Jensen C, Andersen NT, Fleten N, Nielsen CV. Validation of sick leave measures: self-reported sick leave and sickness benefit data from a Danish national register compared to multiple workplace-registered sick leave spells in a Danish municipality. BMC Public Health. 2012;12:661 doi: 10.1186/1471-2458-12-661 ; PubMed Central PMCID: PMC3511193. 22894644 10.1186/1471-2458-12-661 PMC3511193

[17] Svendsen SW, Johnsen B, Fuglsang-Frederiksen A, Frost P. Ulnar neuropathy and ulnar neuropathy-like symptoms in relation to biomechanical exposures assessed by a job exposure matrix: a triple case-referent study. Occup Environ Med. 2012;69(11):773–80. doi: 10.1136/oemed-2011-100499 . 22843442 10.1136/oemed-2011-100499

[18] Moore JS, Garg A. The Strain Index: a proposed method to analyze jobs for risk of distal upper extremity disorders. Am Ind Hyg Assoc J. 1995;56(5):443–58. doi: 10.1080/15428119591016863 . 7754975 10.1080/15428119591016863

[19] Sommer TG, Frost P, Svendsen SW. Combined musculoskeletal pain in the upper and lower body: associations with occupational mechanical and psychosocial exposures. Int Arch Occup Environ Health. 2015;88(8):1099–110. doi: 10.1007/s00420-015-1036-z . 25731853 10.1007/s00420-015-1036-z

[20] Svendsen SW, Frost P, Jensen LD. Time trends in surgery for non-traumatic shoulder disorders and postoperative risk of permanent work disability: a nationwide cohort study. Scand J Rheumatol. 2012;41(1):59–65. doi: 10.3109/03009742.2011.595375 . 22103333 10.3109/03009742.2011.595375

[21] Herup A, Merser S, Boeckstyns M. [Validation of questionnaire for conditions of the upper extremity]. Ugeskr Laeger. 2010;172(48):3333–6. . 21118663

[22] Schønnemann JO, Hansen TB, Søballe K. Translation and validation of the Danish version of the Patient Rated Wrist Evaluation questionnaire. J Plast Surg Hand Surg. 2013;47(6):489–92. doi: 10.3109/2000656X.2013.787934 . 23596992 10.3109/2000656X.2013.787934

[23] Beaton DE, Katz JN, Fossel AH, Wright JG, Tarasuk V, Bombardier C. Measuring the whole or the parts? Validity, reliability, and responsiveness of the Disabilities of the Arm, Shoulder and Hand outcome measure in different regions of the upper extremity. J Hand Ther. 2001;14(2):128–46. . 11382253

[24] Hudak PL, Amadio PC, Bombardier C. Development of an upper extremity outcome measure: the DASH (disabilities of the arm, shoulder and hand) [corrected]. The Upper Extremity Collaborative Group (UECG). Am J Ind Med. 1996;29(6):602–8. doi: 10.1002/(SICI)1097-0274(199606)29:6<602::AID-AJIM4>3.0.CO;2-L . 8773720 10.1002/(SICI)1097-0274(199606)29:6<602::AID-AJIM4>3.0.CO;2-L

[25] MacDermid JC. Development of a scale for patient rating of wrist pain and disability. J Hand Ther. 1996;9(2):178–83. . 8784681 10.1016/s0894-1130(96)80076-7

[26] MacDermid JC, Turgeon T, Richards RS, Beadle M, Roth JH. Patient rating of wrist pain and disability: a reliable and valid measurement tool. J Orthop Trauma. 1998;12(8):577–86. . 9840793 10.1097/00005131-199811000-00009

[27] Ahlstrom L, Grimby-Ekman A, Hagberg M, Dellve L. The work ability index and single-item question: associations with sick leave, symptoms, and health—a prospective study of women on long-term sick leave. Scand J Work Environ Health. 2010;36(5):404–12. . 20372766 10.5271/sjweh.2917

[28] El Fassi M, Bocquet V, Majery N, Lair ML, Couffignal S, Mairiaux P. Work ability assessment in a worker population: comparison and determinants of Work Ability Index and Work Ability score. BMC Public Health. 2013;13:305 doi: 10.1186/1471-2458-13-305 ; PubMed Central PMCID: PMC3637198. 23565883 10.1186/1471-2458-13-305 PMC3637198

[29] Aasheim T, Finsen V. The DASH and the QuickDASH instruments. Normative values in the general population in Norway. J Hand Surg Eur Vol. 2014;39(2):140–4. doi: 10.1177/1753193413481302 . 23520389 10.1177/1753193413481302

[30] Mulders MAM, Kleipool SC, Dingemans SA, van Eerten PV, Schepers T, Goslings JC, et al Normative data for the Patient-Rated Wrist Evaluation questionnaire. J Hand Ther. 2017 doi: 10.1016/j.jht.2017.10.007 . 29132647 10.1016/j.jht.2017.10.007

[31] Lundquist CB, Dossing K, Christiansen DH. Responsiveness of a Danish version of the Disabilities of the Arm, Shoulder and Hand (DASH) questionnaire. Dan Med J. 2014;61(4):A4813 . 24814590

[32] Sorensen AA, Howard D, Tan WH, Ketchersid J, Calfee RP. Minimal clinically important differences of 3 patient-rated outcomes instruments. J Hand Surg Am. 2013;38(4):641–9. doi: 10.1016/j.jhsa.2012.12.032 ; PubMed Central PMCID: PMC3640345. 23481405 10.1016/j.jhsa.2012.12.032 PMC3640345

[33] Gould R, Ilmarinen J, Järvisalo J, Koskinen S, Eds. Dimensions of work ability. Results for the Health 2000 Survey Helsinki: Finnish Centre for Pensions, 2008.

[34] Kinnunen U, Natti J. Work ability score and future work ability as predictors of register-based disability pension and long-term sickness absence: a three-year follow-up study. Scand J Public Health. 2017:1403494817745190. doi: 10.1177/1403494817745190 . 29212430 10.1177/1403494817745190

[35] Biering K, Hjøllund NH, Lund T. Methods in measuring return to work: a comparison of measures of return to work following treatment of coronary heart disease. J Occup Rehabil. 2013;23(3):400–5. doi: 10.1007/s10926-012-9405-x . 23184388 10.1007/s10926-012-9405-x

[36] Kuijer PP, Gouttebarge V, Wind H, van Duivenbooden C, Sluiter JK, Frings-Dresen MH. Prognostic value of self-reported work ability and performance-based lifting tests for sustainable return to work among construction workers. Scand J Work Environ Health. 2012;38(6):600–3. doi: 10.5271/sjweh.3302 . 22538928 10.5271/sjweh.3302

[37] Svendsen SW, Johnsen B, Fuglsang-Frederiksen A, Frost P. Prognosis of ulnar neuropathy and ulnar neuropathy-like symptoms in relation to occupational biomechanical exposures and lifestyle. Scand J Work Environ Health. 2013;39(5):506–14. doi: 10.5271/sjweh.3352 . 23429966 10.5271/sjweh.3352

[38] Schouten LS, Bültmann U, Heymans MW, Joling CI, Twisk JW, Roelen CA. Shortened version of the work ability index to identify workers at risk of long-term sickness absence. Eur J Public Health. 2016;26(2):301–5. doi: 10.1093/eurpub/ckv198 . 26498956 10.1093/eurpub/ckv198

[39] Lundin A, Leijon O, Vaez M, Hallgren M, Torgen M. Predictive validity of the Work Ability Index and its individual items in the general population. Scand J Public Health. 2017;45(4):350–6. doi: 10.1177/1403494817702759 . 28385066 10.1177/1403494817702759

[40] Ahlstrom L, Hagberg M, Dellve L. Workplace rehabilitation and supportive conditions at work: a prospective study. J Occup Rehabil. 2013;23(2):248–60. doi: 10.1007/s10926-012-9391-z ; PubMed Central PMCID: PMCPMC3666126. 23065193 10.1007/s10926-012-9391-z PMC3666126

[41] Rubak TS, Svendsen SW, Søballe K, Frost P. Total hip replacement due to primary osteoarthritis in relation to cumulative occupational exposures and lifestyle factors: a nationwide nested case-control study. Arthritis Care Res (Hoboken). 2014;66(10):1496–505. doi: 10.1002/acr.22326 . 24664794 10.1002/acr.22326

[42] Dinah AF, Vickers RH. Smoking increases failure rate of operation for established non-union of the scaphoid bone. Int Orthop. 2007;31(4):503–5. doi: 10.1007/s00264-006-0231-7 ; PubMed Central PMCID: PMC2267625. 16947049 10.1007/s00264-006-0231-7 PMC2267625

[43] Little CP, Burston BJ, Hopkinson-Woolley J, Burge P. Failure of surgery or scaphoid non-union is associated with smoking. J Hand Surg Br. 2006;31(3):252–5. doi: 10.1016/j.jhsb.2005.12.010 . 16488521 10.1016/j.jhsb.2005.12.010

[44] Scolaro JA, Schenker ML, Yannascoli S, Baldwin K, Mehta S, Ahn J. Cigarette smoking increases complications following fracture: a systematic review. J Bone Joint Surg Am. 2014;96(8):674–81. doi: 10.2106/JBJS.M.00081 . 24740664 10.2106/JBJS.M.00081

[45] Hernandez RK, Do TP, Critchlow CW, Dent RE, Jick SS. Patient-related risk factors for fracture-healing complications in the United Kingdom General Practice Research Database. Acta Orthop. 2012;83(6):653–60. doi: 10.3109/17453674.2012.747054 ; PubMed Central PMCID: PMC3555441. 23140093 10.3109/17453674.2012.747054 PMC3555441

[46] Lee RJ, Hsu NN, Lenz CM, Leet AI. Does obesity affect fracture healing in children? Clin Orthop Relat Res. 2013;471(4):1208–13. doi: 10.1007/s11999-012-2626-7 ; PubMed Central PMCID: PMC3586024. 23054519 10.1007/s11999-012-2626-7 PMC3586024

